# MicroRNA Let-7adf in Tet regulation

**DOI:** 10.18632/aging.102101

**Published:** 2019-07-16

**Authors:** Shuai Jiang

**Affiliations:** 1 Division of Biology and Biological Engineering, California Institute of Technology, Pasadena, CA 91125, USA

**Keywords:** Tet, MicroRNA Let-7adf, H19, Lin28a, myeloid cell

In our recent work [1], we examined let-7adf to understand pathways that a cell uses to respond to inflammatory signaling. We know myeloid cells are critical to Tet2-mediated IL-6 inhibition. The action of Tet2 is in myeloid cells because a conditional KO and a global KO of Tet2 have similar effects. Our work adds to the previous work [2], which revealed that myeloid cell-specific Tet2-deficient mice were more susceptible to dextran sulfate sodium (DSS)-induced colitis and displayed a more severe inflammatory phenotype than WT mice, with exacerbated colon inflammation including higher production of IL-6.

In this work, we discovered a novel regulatory control of Tet2 by let-7adf. Although functional roles of Tet2 in macrophages have been extensively studied, the knowledge of how Tet2 is precisely regulated has been limited. The questions regarding the complicated network of regulation of Tet2 through interaction with the let-7 miR remain open and will be an area of significant interest. The posttranscriptional effectors engaged in the regulation of gene expression, including RNA-binding proteins and other non-coding RNAs, can affect miR-mediated gene regulation. For instance, Lin28 RNA binding protein blocks biogenesis of let-7 miRs [3] and the H19 long non-coding RNA acts as a molecular sponge for the let-7 family of miRs [3]. We would speculate that either Lin28 or H19 might control Tet2 through regulating behavior of let-7 in activated macrophages.

Of the 3 *Tet* genes, we found that the let-7adf cluster specifically targets only Tet2 in macrophages, although both *Tet2*and *Tet3* gene have potential binding sites for let-7 in their 3’UTRs. Given the complexity and degree of interaction between miRs and predicted putative target genes, to understand how this miR cluster achieves its specificity to potential target genes is important to identifying its role in different cellular contexts. It will be important to examine whether this let-7 miR cluster regulates Tet2 and Tet3 in different types of immune cells.

Deletion of Tet2 in mice is sufficient to cause myeloid malignancies [4], to predispose to mature B-cell malignancies [5] and to lead to hyper-mutagenicity in haematopoietic stem cells [6]. Tet2 is also an important regulator of CD8+ T cell fate decisions [6,7]. We understand the interaction of Tet2 with the let-7adf cluster in combating bacterial infection, but this miR cluster may regulate Tet2 as its target in other physiological and pathological circumstances through the dual-mechanisms that we report here. To more precisely understand the physiological or pathological roles of the let-7adf cluster/Tet2 axis in other immune cells— for instance T cells—will require further dissection of roles of let-7adf/Tet2 in specific immune tissues by using conditional engineered mouse models.

LPS-induced TLR signaling must be tightly controlled to avoid excessive inflammation and a return to homeostasis after infection. The significance of LPS-activated TLR signaling has focused attention on the relevant regulatory mechanisms over the last few years [8]. Although miRs are critical regulators of macrophage inflammatory responses, whether and how they are involved in LPS-induced signaling pathway in murine macrophages needs to be fully understood. We have utilized miR engineered mouse models to infer the regulatory mechanisms of the miR cluster on LPS-induced signaling pathways. It would be worthy to investigate the physiological and/or pathological role of other let-7 clusters such as let-7bc cluster in counteracting with various types of bacterial infection using engineered mouse models in near future. These future works may provide a deeper understanding of single microRNA let-7 clusters in myeloid-cell biology.
